# Pneumonia panel results and antibiotic prescribing in COVID-19 patients in 2020 versus 2022

**DOI:** 10.1017/ash.2023.271

**Published:** 2023-09-29

**Authors:** Aysha Hussain, Trevor Van Schooneveld, Scott Bergman, Molly Miller, Paul D. Fey, Erica Stohs

## Abstract

**Background:** Antibiotics are frequently prescribed in patients with COVID-19 infections to treat secondary bacterial pneumonia. The pneumonia panel (PNP) is a molecular diagnostic tool that rapidly detects 33 bacterial and viral targets. The utility of this panel in COVID-19 patients and how it may direct antibiotic use is unknown. We sought to understand the utilization of PNP in patients with COVID-19 pneumonia over time by comparing clinical parameters, microbiologic results, and antibiotic use between May–December 2020 and January–July 2022. **Methods:** We implemented the PNP in May 2020 with antimicrobial stewardship guidance, provider education, and order restriction to critical care and infectious disease clinicians. From February–July 2021 prescribers received regular structured antimicrobial stewardship feedback regarding PNP results; from August 2021 to January 2022, no antimicrobial stewardship feedback was provided; from February to July 2022, intermittent feedback was provided. We compared PNP and culture results from sputum or bronchoalveolar lavage samples and antibiotic use and modification within 24 hours of PNP result from patients with confirmed COVID-19 pneumonia between May–December 2020 and January–July 2022. Clinical data and antibiotic use were abstracted through chart review. We excluded patients who died within 72 hours of PNP, those who had concurrent nonpulmonary infections, and those whose COVID-19 test was >30 days prior. **Results:** We included 114 patients in 2020 and 71 patients in 2022. The overall median age was 61 years, 71% were male, and 66% were mechanically ventilated without statistical differences between the cohorts, including their comorbidities. Acute or worsening hypoxia remained the predominant indication for PNP (77% in 2020 vs 75% in 2022, NS). The median number of days between admission and PNP was 4 (IQR, 1–8) in 2020 versus 3 (IQR, 1–7), and the difference was not significant. PNP and culture results in Table 1 show that *Staphylococcus aureus* and *Hemophilus influenzae* were the pathogens most commonly identified. Table 2 describes empiric prescribing and modifications for commonly prescribed antibiotics. Prescribers used empiric cefepime and ceftriaxone more in 2020 and vancomycin more in the 2022 group; however, these were not statistically significant. Cefepime de-escalation was more common in 2022 (53% vs 28%; *P* = .03). Antibiotic modifications within 24 hours of PNP remained similar in 2020 vs 2022. Although vancomycin cessation was more common in 2020 (78%) versus 2022 (57%), the difference was not statistically significant. **Conclusions:** With ASP guidance, PNP may be a useful tool to stop or target antibiotics for secondary bacterial pneumonia in COVID-19 pneumonia. Early vancomycin cessation (prior to culture results) may be an enduring consequence of PNP implementation.

**Figure:**
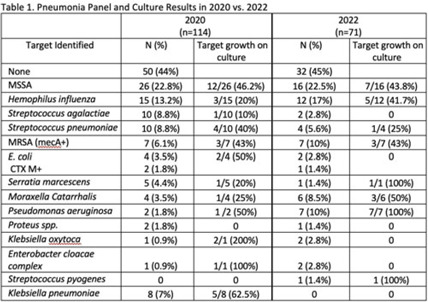

**Figure:**
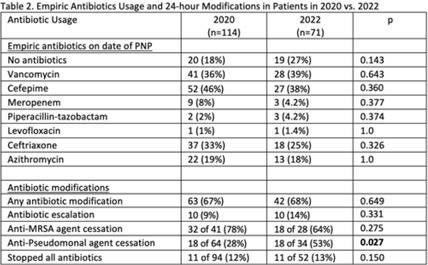

**Disclosures:** None

